# Primary malignant PEComa of the mandible. Report of an unusual case

**DOI:** 10.4317/jced.58347

**Published:** 2021-09-01

**Authors:** Lourdes Deutor-Garcia, Clara Chamorro-Santos, Lydia Fraile-Ruiz, Jose-Juan Pozo-Kreilinger, Hernández-Vila Cristina

**Affiliations:** 1 Degree in Medicina, Department of Oral and Maxillofacial Sugery. Hospital Universitario Virgen de las Nieves, Granada; 2 Degree in Medicine, Department of Pathological Anatomy Hospital Universitario Virgen de las Nieves, Granada (España); 3 Degree in Medicine. Department of Pathological Anatomy. Hospital Universitario La Paz, Madrid (España)

## Abstract

Malignant PEComa is a rare entity that usually origins at visceral, retroperitoneal and abdominopelvic sites. In the present paper, we describe an extremely unusual manifestation of malignant PEComa involving the mandible in a 48 years-old female patient focusing on the anatomopathological findings and differential diagnosis. The therapeutic management based on the head and neck tumor board indications is also discussed.

** Key words:**Malignant PEComa, PEComa of the mandible, PEComa pathology, Oral cavity unusual neoplasm.

## Introduction

PEComas (Perivascular Epithelioid Cells differentiation tumor) are a rare and anatomically ubiquitous family of mesenchymal neoplasms composed of nests and sheets of epithelioid cells with clear to granular eosinophilic cytoplasm and focal association with blood vessel walls (1). Its origin is muscle/melanocytic, so the expression of both smooth muscle and melanocytic markers is observed. Furthermore, PEComas expression of CD68, CD63, cathepsin K and TFE3 has been reported. Epithelial markers are not found in the immunohistochemical (IHC) analysis (2,3).

Most PEComas are benign. Malignant PEComas present an aggressive behavior, with potential for local recurrences, metastasis and death (4). The presence of high nuclear grade, infiltrative grown pattern, high cellularity, necrotic areas, vascular invasion, mitotic activity > 1mytosis/50 HPF and tumor size > 5cm are characteristics of malignant lesions (5).

PEComas presentation as a primary bone lesion is extremely rare. To our knowledge, only 11 cases of primary bone origin have been reported in the literature (excluding mandible). Pain is the most frequent clinical sign; swallowing and functional impotence are also common and a pathological fracture might happen if the osteolysis weakens the bone enough. Complementary image studies usually show an osteolytic lesion that associates a soft tissue mass and destruction of cortical bone in cases of high aggressivity (6).

According to the therapeutic guidelines, surgical resection of primary tumor and its recurrences is the first option. Second therapeutic step involves adjuvant treatment with mTOR inhibitors, particularly in tumors with evidence of mTORC1 activation and somatic deletion of TSC1(7).

## Case Report

We report the case of a 48-year-old female patient without relevant medical history, referred to our department (Oral and Maxillofacial Surgery department, Hospital Universitario Virgen de las Nieves, Granada) for the evaluation of a fast-growing and hyperemic surface soft tissue mass that involved the alveolar ridge of the left mandible. Specifically, the lesion had grown in the last two weeks. Pain and swallowing were the main symptoms; furthermore, the patient did not experiment inferior alveolar nerve paresthesia. The major diameter of the lesion was 4cm and mobility of dental pieces 37 and 38 was reached in association. The clinical evaluation of the neck was negative.

**Figure 1:**
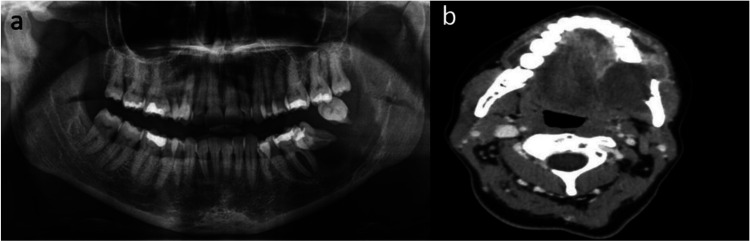
Diagnostic imaging techniques. a) OPG. b) CT scan axial view.

An Orthopantomography (OPG) and a Computed Tomography (CT) scan were performed as diagnostic imaging techniques. The OPG evidenced erosion of the cortical bone subjacent to pieces 37 and 38 and the CT scan showed a tumor with smooth contour of approximately 27x40x47 cm involving left angle of mandible; not cervical dissemination was found (Fig. 1).

**Figure 2:**
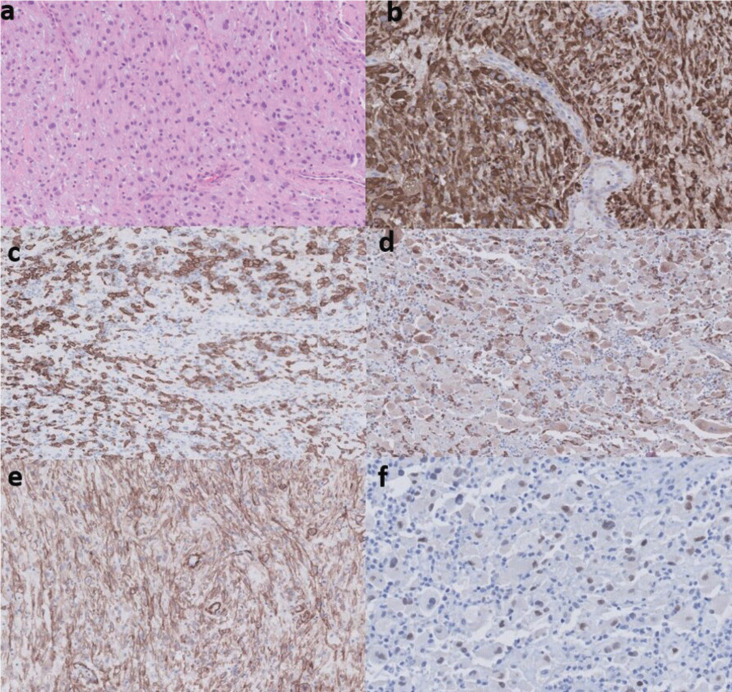
Anatomopathological findings. a) HE technique (x40). Epithelioid cells with an eosinophilic, clear and granular cytoplasm and inflammatory cells. Diffuse growth pattern. b) IHC technique. CD63* expression (immunoperoxidase x40). c) IHC technique. CD163* expression (immunoperoxidase x40). d) IHC technique. Cathepsin K* expression (immunoperoxidase x40). e) IHC technique. KBA 62** expression (immunoperoxidase x40). f) IHC technique. TFE3 expression (immunoperoxidase x40). *histiocytic marker **melanocytic marker

As first therapeutic approach, a conservative resection of the tumor was performed. The sample was analyzed by the Pathological Anatomy department of our institution in collaboration with the Pathological Anatomy department of the Hospital Universitario La Paz, Madrid. The anatomopathological analysis evidenced morphologic characteristics compatible with several entities, discarded based on different criteria. Conventional hematoxylin-eosin (HE) technique showed a neoplasm with diffuse growth pattern composed of epithelioid cells with an eosinophilic, clear and granular cytoplasm and inflammatory cells. The IHC showed positivity for histiocytic markers CD68, CD63, CD163 and cathepsin K. The muscle marker SMA positivity was quite focal. TFE3, MITF1 and the melanocytic marker KBA.62 turned out also positive. All the findings were compatible with PEComa (Fig. 2).

**Figure 3:**
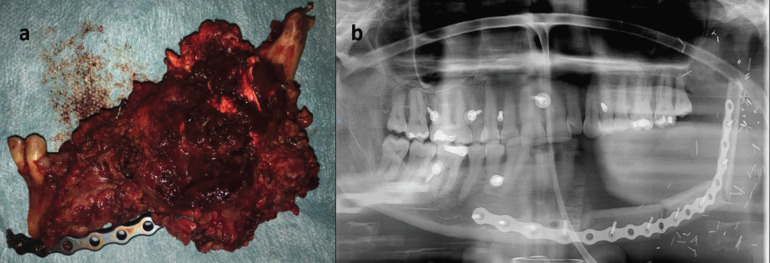
a) Surgical specimen (hemimandibulectomy of the left mandible). b) OPG after fibula free flap reconstruction.

Once the definitive diagnosis was available, an oncological resection of the tumor accompanied by left neck dissection were performed. The resection included a segmental mandibulectomy, so the mandible was reconstrued with an osteocutaneous fibula free flap (Fig. 3). The anatomopathological analysis of the surgical specimen evidenced a tumor size of 8x5x4.5cm. The neoplasm was in contact with the surgical margins and muscle invasion was found in both sternocleidomastoid and medial pterygoid muscles. Metastatic involvement of the neck was reached in Level Ib, Level IIa and Level IIb.

As for malignant characteristics, in this case, the tumor size was higher than 5cm and the lesion showed infiltrative grown pattern, high nuclear grade, 6 mitosis/10HPF and necrosis.

Given the malignant behavior of the neoplasm, the impossibility of achieve a margin-negative resection (R0) and the metastatic involvement of the neck, adjuvant treatment with Everolimus was administrate. Consequently, a partial response was achieved during seven months. However, when the adjuvant treatment ended, the patient suffered tumor persistence. Thus, palliative radiation therapy was administrated as a last therapeutic option, according to the consensus of the Head and Neck tumor board of our Hospital. Unfortunately, the patient died twenty months after the diagnosis as a cause of airway obstruction.

## Discussion

The bone is rarely affected by PEComas and the mandible is an extremely unusual primary tumor location. To our knowledge, only one single case of malignant PEComa of the mandible has been reported previously in a 77 years-old female patient. The therapeutic management elected was the surgical resection. A segmental mandibulectomy was performed and the mandible was reconstructed with a prebent reconstruction plate. Adjuvant radiation therapy was posteriorly administered. However, MTOR inhibitors were not administered. The patient did not experiment any loco-regional recurrence up to 2 years after surgery (5).

The differential diagnosis was complex due to the histological heterogeneity of the piece. The nuclear

expression of TFE3 has been described in a limited number of entities (8) that share morphologic characteristics: renal metastasis, particularly translocation renal cell carcinoma, were discarded due to the absence of renal tumors and the negativity in the epithelial markers CKAE1/AE3 (9). The absence of distinctive features of alveolar sarcoma dismisses this entity and clear cell sarcoma doesn´t express TFE3 (10). S100 protein in IHC analysis resulted also negative, so the diagnosis of granular cell tumor, was also rejected (11).

Other neoplasm with TFE3 as the epithelioid hemangioendothelioma was discarded due to the absence of distinctive morphologic characteristics and the negativity of CD34/CK (12).

The histiocytic-like morphology of the cells and the expression of histiocytic markers have led to the wrong classification of the PEComas as tumors of histiocytic origin. However, lymphocytes plasma cells and or eosinophiles did not intermix in the lesion (as it happens in histiocytic sarcomas) and protein s100 and CD1a were negative (markers proper of Langerhans cells histiocytosis). Moreover, in the histiocytic lesions TFE3 positivity is not described (13). The main muscle/melanocytic markers defining of the PEComas were absent. Nevertheless, the absence of melanocytic markers with expression of TFE3 and focal expression of SMA combined with the characteristic morphology of this lesions is described (14).

In regard to the therapeutic management, the surgical resection wasn´t effortless. The malignant behavior of the neoplasm and its fast growth led to an extensive locoregional infiltration, so the accomplishment of a R0 surgery was not possible. For this reason, the administration of MTOR inhibitors was justified as second therapeutic step (7,15). Although the response of malignant PEComas to the radiation therapy is not demonstrated, the limited response to MTOR inhibitors and the tumor persistence, justified its use as empirical approach for the management of this extremely uncommon disease.
